# Simultaneous treatment for *Mycobacterium-avium* complex lung disease and allergic bronchopulmonary aspergillosis: A case report

**DOI:** 10.1016/j.rmcr.2021.101488

**Published:** 2021-07-24

**Authors:** Nobuhiro Oda, Kei Nakashima, Yuya Homma, Norihiko Kubota, Jumpei Taniguchi, Tatsuya Nagai, Yoshinori Yoshimi, Ayumu Otsuki, Hiroyuki Ito

**Affiliations:** a Department of General Internal Medicine, Kameda Medical Center, Kamogawa, Japan; b Department of Pulmonology, Kameda Medical Center, Kamogawa, Japan

**Keywords:** *Mycobacterium-avium* complex, Allergic bronchopulmonary aspergillosis, Therapy

## Abstract

Recently, there are several reports of simultaneous allergic bronchopulmonary aspergillosis (ABPA) and *Mycobacterium-avium complex* (MAC) lung disease. However, the strategies for early diagnosis and appropriate treatment for patients with both ABPA and MAC lung disease have not been established. Here, we report a case with ABPA complicated by MAC lung disease, which was successfully diagnosed and treated by simultaneous administration of systemic steroids and antimycobacterial drugs. Bronchoscopy can be useful in the diagnosis of such cases. Furthermore, in a patient with concurrent ABPA and MAC lung disease, simultaneous treatments for both diseases could reduce both diseases.

## 1 Introduction

Allergic bronchopulmonary aspergillosis (ABPA) is a complex hypersensitivity reaction in response to the colonization of the airways with *Aspergillus fumigatus* [1]. It is often complicated by asthma and cystic fibrosis, causing recurrent attacks that cause lung fibrosis, leading to respiratory failure [1]. Systemic glucocorticoids are the first line of treatment to control the symptoms and limit progressive lung injury [1].

The number of patients with pulmonary diseases caused by non-tuberculous mycobacteria (NTM), such as *Mycobacterium-avium* complex (MAC), has been increasing worldwide in recent years [2,3]. Treatment initiation time is crucial because some patients with MAC lung disease exhibit progressive pulmonary destruction, even leading to death [4]. Several case reports have shown that patients with MAC lung disease rarely develop ABPA [[5], [6], [7], [8], [9], [10]]. Since the radiological findings of ABPA and MAC lung disease are similar as both show bronchodilation, the differential diagnosis between these two diseases can be challenging [1,11]. However, the strategies for early diagnosis and appropriate treatment for patients with both ABPA and MAC lung disease have not been established because current evidence in this field is limited.

We report a case with ABPA complicated by MAC lung disease, which was successfully diagnosed and treated by simultaneous administration of systemic steroids and antimycobacterial drugs.

### 1.1 Case presentation

**Fig. 1:**
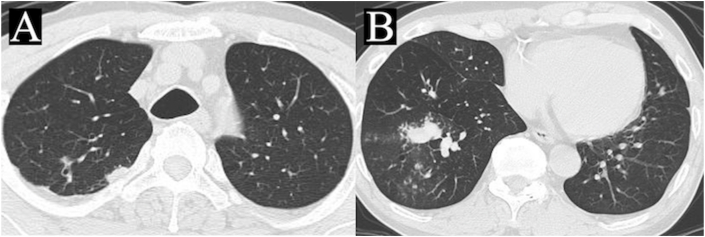
At the first visit to a primary care doctor, chest CT showed the new infiltrations in the upper (A) and lower lobes (B) of the right lung. CT: computed tomography.

A 70-year-old male Japanese with a history of rheumatoid arthritis (RA) was referred to the outpatient department of pulmonology because of non-resolving infiltration on chest imaging. He was diagnosed with RA 9 years ago, which was stable with iguratimod 100 mg per day, prednisolone (PSL) 0.5 mg per day, and methotrexate 7 mg per week. With respect to the timing of the diagnosis, pulmonary diseases associated with RA, such as bronchitis or interstitial pneumonia, were not detected on chest radiography or computed tomography (CT). Two months ago, he visited a primary care doctor with a chief complaint of cough for 3 months. He was diagnosed with community-acquired pneumonia based on the new nodules in the upper and lower lobes of the right lung, and amoxicillin-clavulanic acid combination was administered for 14 days (Fig. 1A and B). However, the abnormal infiltration on chest CT worsened 1 month after treatment. Another lung disease, such as organizing pneumonia or tuberculosis, was suspected based on the clinical course and chest CT findings and was referred to our department.

**Fig. 2:**
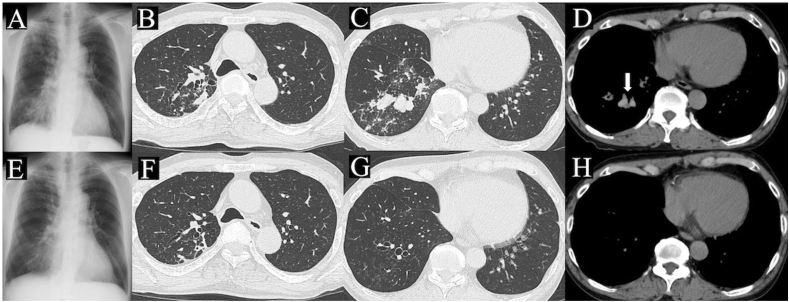
Chest radiograph of the patient, acquired at the initial visit to our hospital, showing consolidations in the right upper and lower pulmonary fields (A). Chest CT scan of the patient showing worsening consolidations in the upper lobe of the right lung (B), central bronchiectasis, and centrilobular nodules in the lower lobe of the right lung (C). HAM suggests allergic bronchopulmonary aspergillosis evident in the lower lobe of the right lung (D) (arrow). Chest radiograph acquired one month after treatment initiation shows disappearance of consolidations in the right upper and lower pulmonary fields (E). Chest CT image showing a reduction in consolidations and nodules in the upper (F) and lower lobes of the right lung (G). HAM in the lower lobe of the right lung disappeared (H). CT: computed tomography; HAM: high attenuation mucus.

At the patient's initial visit to our department, his height was 1.60 m, weight 54 kg, temperature 36.3°, blood pressure 156/76 mm Hg, pulse 92/min, respiratory rate 16/min with an O_2_ saturation of 95 % on room air, and his consciousness was lucid. Neither wheeze nor crackle was audible on chest auscultation. Laboratory tests showed a white blood cell count of 9000/mm^3^ with eosinophilia (3825/mm^3^), C-reactive protein level of 0.34 mg/dL, and negative *anti*-GLP core IgA antibody test (Table 1). Chest radiographs showed abnormal infiltration in the upper and lower fields of the right lung (Fig. 2A). Chest CT showed a nodular shadow in the apex of the right lung, central bronchiectasis, and centrilobular nodules in the lower lobe of the right lung (Fig. 2B and C). In addition, chest CT showed bronchiectasis with mucus plugs, called high attenuation mucus (HAM), which have a density equal to or greater than that of the paraspinal muscles in the mediastinal window setting (Fig. 2D).

**Table 1:** Laboratory test results of the patient.

Hematology			Biochemistry					
White blood cells	90	× 10^2^/μL	Total protein	6.9	g/dL	Na	140	mEq/L
Neutrophils	37.6	%	Albumin	4.2	g/dL	K	4.5	mEq/L
Eosinophils	42.5	%	AST	19	U/L	Cl	103	mEq/L
	3825	/uL	ALT	18	U/L			
Basophils	0.7	%	LDH	170	U/L	β-D-glucan	<0.5	pg/mL
Monocytes	3.9	%	ALP	353	U/L	GPL core-IgA	negative	
Lymphocytes	15.3	%	γ-GTP	18	U/L	IGRA	negative	
Red blood cells	505	× 10^4^/μL	T-Bil	0.9	mg/dL	*A. fuigatus* IgG	4	titer
Hemoglobin	15.8	g/dL	BUN	14	mg/dL	IgE	233	IU/mL
Hematocrit	47.2	%	sCre	0.66	mg/dL	*A. fuigatus* IgE	2.19	UA/mL
Platelet count	15.2	× 10^4^/μL	CRP	0.34	mg/dL			

AST: aspartate aminotransferase, ALT: alanine transaminase, LDH: lactate dehydrogenase, ALP: alkaline phosphatase, γ-GTP: gamma-glutamyl transpeptidase, T-Bil: total bilirubin, BUN: blood urea nitrogen, sCre: serum creatinine, CRP: c-reactive protein, IGRA: interferon-gamma release assay.

Differential diagnoses included ABPA, airway disease associated with RA, organizing pneumonia, eosinophilic pneumonia, and mycobacterial infection. Three consecutive sputum smears were negative for mycobacteria; bronchoscopy was performed, which revealed the presence of thick sputum filling the lumen of bronchi in the lower lobe of the right lung with findings suggestive of mucoid impaction. Bronchial washing and direct biopsy by forceps of mucus obtained from the bronchial mucosa were performed. Cytology of the bronchoscopy specimen revealed acid-fast bacteria using Ziehl–Neelsen staining and a Y-shaped, Grocott stain-positive fungus, possibly *Aspergillus*. Histological examination of the mucous membrane around the mucoid impaction revealed eosinophilic inflammation of the bronchial mucosa, consistent with ABPA. Serum total IgE level was 233 IU/mL; however, the results of *Aspergillus fumigatus*–specific IgE and IgG tests were positive (Table 1). Fungal cultures were negative, while mycobacterial cultures returned positive for M. *avium*. Based on clinical symptoms combination, bronchoscopic luminal findings, and laboratory and imaging studies, the patient was diagnosed with ABPA and MAC lung infection. While this patient's case did not meet the International Society for Human and Animal Mycology diagnostic criteria for ABPA; however, it met the Japanese criteria [12,13]. We believed that the negative *anti*-GLP core IgA antibody test (Table 1) result was a false negative given that the patient was using steroids and his MAC lung disease was mild.

Pulmonary function tests were performed: vital capacity 2.56L (79.3 % of predicted) and forced expiratory volume in 1 second (FEV1) 80.3 % suggestive of a restrictive ventilation disorder. Airway reversibility test using a short-acting beta-2 agonist showed marginal bronchodilator response (FEV1.0) did not improve more than 12 % (10.2 %), which amounted to 200 mL. The level of nitric oxide in the exhaled air was 48 ppb. From these findings, the presence of decreased respiratory function and an asthmatic status was considered.

**Fig. 3:**
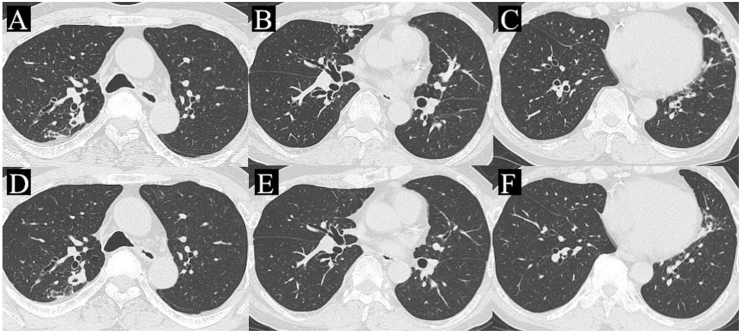
Chest CT, acquired three months after the discontinuation of treatment, demonstrates the increased nodular shadow in the upper lobe (A) and the newly appeared, bilateral, centrilobular nodules in the lingular segments (B, C). After four months of clarithromycin and rifampicin administration, these improved (D–F). CT: computed tomography.

We administered ethambutol (750 mg/day), clarithromycin (800 mg/day), and rifampicin (450 mg/day) for MAC lung disease treatment because of the progressive worsening of the nodular lesions on chest CT and the risk of exacerbation of MAC lung disease owing to high-dose corticosteroid therapy for ABPA. In addition, we started a systemic corticosteroid (PSL 50 mg/day) for ABPA treatment. Initially, we considered a 25 mg/day PSL dose (0.5 mg/kg); however, we decided to administer 50 mg/day because rifampicin decreases the serum concentration of PSL. Although eosinophil count was restored on the seventh day after treatment initiation, respiratory symptoms such as cough persisted. Thus, we started inhalational vilanterol/fluticasone furoate (VI/FF), one puff per day (VI 25 μg and FF 100 μg/day). The respiratory symptoms gradually improved after the initiation of inhalational VI/FF. After one month, the nodular lesions, consolidation, and mucoid impaction showed significant improvement on chest radiographs (Fig. 2E) and CT (Fig. 2F, G, and H). However, the patient developed a fever one month after initiating treatment. Drug fever was immediately suspected, and his antimycobacterial drugs were discontinued with subsequent resolution of the fever within 7 days. Nevertheless, he developed fever again after re-administering ethambutol (500 mg), and we consequently discontinued administering clarithromycin and ethambutol. Thereafter, the patient was treated with PSL alone because his symptoms and CT findings were stable. The PSL dose was gradually tapered and discontinued for three months. However, three months after discontinuing treatment, the patient visited the outpatient department presenting with cough and dyspnea. Laboratory test results revealed eosinophilia (eosinophil count 1383/μL). Chest CT showed an increased nodular shadow in the upper lobe and newly appeared bilateral, centrilobular nodules in the lingular segments (Fig. 3A–C). The administration of clarithromycin (800 mg), rifampicin (450 mg), and PSL (20 mg) was initiated. After one month, his symptoms and eosinophilia improved, and after four months, the new lesions on chest CT improved (Fig. 3D–F). Antimycobacterial drugs will be continued for at least 12 months, and PSL will be given for about 3–4 months according to the symptoms.

## 2 Discussion

We successfully diagnosed and treated ABPA complicated by MAC lung disease during the treatment of RA. The clinical course of this patient provided two important clinical suggestions that when ABPA is suspected, the possibility of concomitant MAC lung disease should also be considered at the time of initial diagnosis and that concurrent treatment should be considered when both ABPA and MAC lung diseases are detected.

Several reports have documented the co-occurrence of MAC lung disease and chronic pulmonary aspergillosis [14,15]. A retrospective study showed that pulmonary ABPA occurred in 16.7 % (5/30) patients during the treatment of MAC lung disease [14]. Another study reported that 4.6 % (5/109) MAC lung disease patients were diagnosed with ABPA [15]. One report has shown that 16 % of 42 patients with allergic bronchopulmonary mycosis (ABPM) developed MAC lung disease during treatment [16]. According to this study, 6 of 7 patients with ABPM (85.7 %) received steroid treatments before NTM lung disease onset, and 20 of 35 patients with ABPM without NTM lung disease (57.1 %) received steroids, suggesting an association between steroid use and NTM lung disease. Previous retrospective studies have shown that the risk of developing MAC lung disease increases with steroid dose. A previous Taiwanese study showed an increased risk of MAC lung disease development by higher amounts of steroids used to treat RA [17]. In addition, another study suggests that oral steroids may be a risk factor for resistance pulmonary MAC lung disease treatment [18]. In fact, a case report documented death due to MAC lung disease re-activation by ABPA treatment [5]. In this case, the patient was diagnosed with ABPA complicated MAC lung disease when taking iguramimod 100 mg per day, PSL 0.5mg per day, and methotrexate 7mg per week for RA treatment.

ABPA and NTM lung disease are frequently associated, but their differential diagnosis is difficult. Both ABPA and MAC lung disease may show a “tree-in-bud” appearance on CT, which may further render the distinction between them difficult [1,12]. A report of a patient with cough, in whom a tree-in-bud appearance without asthma symptoms was treated as NTM lung disease, found ABPA diagnosed in the end; therefore, NTM was colonized [6]. In addition, Aspergillus precipitating antibodies are useful in the diagnosis of ABPA and are included in the diagnostic criteria. However, in the study mentioned above, 18.3 % of patients with MAC lung disease were positive for Aspergillus antibodies. Only 25 % were diagnosed with ABPA; thus, serological tests are not sufficiently effective [15]. In this case, an increase in blood eosinophils, “tree-in-bud” appearance, and HAM on CT were suggestive of ABPA; however, the patient was diagnosed with ABPA and MAC lung disease based on the findings on bronchoscopy. Therefore, we believe that bronchoscopy may be a useful tool to examine patients with ABPA and MAC lung disease.

Another important point of our case is that concurrent treatment should be considered when co-infection of *Aspergillus* and NTM is found. For example, Kadamkulam et al. reported a case in which ABPA developed during treatment of MAC disease, treatment was changed, and systematic steroid was started simultaneously with good results [7]. Conversely, Mussaffi et al. reported 6 cases of the clinical worsening of NTM with ABPA during systemic steroid therapy [8]. In this case, when ABPA and MAC lung diseases were diagnosed, we considered the possibility of an exacerbation. Therefore, we treated both ABPA and MAC lung disease simultaneously, resulting in the remission of both diseases. Recently, ABPA patients treated with molecular targeted agents without NTM exacerbation have been reported, and further studies on the treatment regimen of ABPA complicated by MAC lung disease are expected [9,10].

## 3 Conclusion

The possibility of ABPA complicated by pulmonary MAC disease should be considered. Moreover, bronchoscopy can be useful to diagnose such cases. In a patient with concurrent ABPA and MAC lung disease, simultaneous treatments for both diseases could result in the remission of both diseases.

## Funding

None.

## Declaration of competing interest

The authors declare that they have no known competing financial interests or personal relationships that could have appeared to influence the work reported in this paper.
